# Supplementary material for: Resolving the full spectrum of human genome variation using Linked-Reads
Source: Genome Res. 2019 Apr;29(4):635–45. doi: 10.1101/gr.234443.118 (PMC6442396; doi:10.1101/gr.234443.118)
Supplement: Supplemental Material [file supp_gr.234443.118_Supplemental_longranger-2.2.zip › longranger-2.2.tar.bz2!/lr2.2-public/lib/stan/cmdstan/stan/doc/api/html/math_2matrix_2crossprod_8hpp_source.html]

Stan: src/stan/math/matrix/crossprod.hpp Source File


|  |  |
| --- | --- |
|  | Stan  2.5.0  probability, sampling & optimization |


- Main Page
- Related Pages
- Namespaces
- Classes
- Files

- File List
- File Members

All Classes Namespaces Files Functions Variables Typedefs Enumerations Enumerator Friends Macros Pages

- src
- stan
- math
- matrix

crossprod.hpp

Go to the documentation of this file.

1 #ifndef STAN\_\_MATH\_\_MATRIX\_\_CROSSPROD\_HPP

2 #define STAN\_\_MATH\_\_MATRIX\_\_CROSSPROD\_HPP

3

4 #include <stan/math/matrix/typedefs.hpp>

5 #include <stan/math/matrix/tcrossprod.hpp>

6

7 namespace stan {

8  namespace math {

9

16  inline matrix\_d

17  crossprod(const matrix\_d& M) {

18  return tcrossprod(M.transpose());

19  }

20

21  }

22 }

23 #endif

typedefs.hpp

stan::math::tcrossprod

matrix\_d tcrossprod(const matrix\_d &M)

Returns the result of post-multiplying a matrix by its own transpose.

**Definition:** tcrossprod.hpp:17

stan::math::matrix\_d

Eigen::Matrix< double, Eigen::Dynamic, Eigen::Dynamic > matrix\_d

Type for matrix of double values.

**Definition:** typedefs.hpp:23

tcrossprod.hpp

stan::math::crossprod

matrix\_d crossprod(const matrix\_d &M)

Returns the result of pre-multiplying a matrix by its own transpose.

**Definition:** crossprod.hpp:17


---

[ Stan Home Page ]

*© 2011–2014,
Stan Development Team.*
